# Giant lipoma with an unusual anatomical location successfully managed in a resource-limited setting: a case report

**DOI:** 10.1093/jscr/rjag074

**Published:** 2026-02-17

**Authors:** Wondwosen Mengist Dereje, Desalegn Kefale Aegash, Alem Demissie Bogale, Mengist Asmamaw Tegegne

**Affiliations:** Department of Neurology, University of Gondar College of Medicine and Health Sciences, Gondar, 196, Ethiopia; Department of Surgery, University of Gondar College of Medicine and Health Sciences, Gondar, 196, Ethiopia; Department of Pathology, University of Gondar College of Medicine and Health Sciences, Gondar, 196, Ethiopia; Department of Surgery, University of Gondar College of Medicine and Health Sciences, Gondar, 196, Ethiopia

**Keywords:** giant lipoma, benign tumor, FNAC, surgical excision, case report

## Abstract

Lipomas are benign tumors arising from abnormal proliferation of adipocytes, commonly found in fat-rich regions, though rarely in the head and neck. They usually occur between 40 and 50 years of age, with a slight male predominance. This report presents a 29-year-old male with a giant posterior neck lipoma, emphasizing the need to distinguish it from soft tissue malignancy. The swelling began as a small lump and gradually enlarged to 20 × 25 cm over three years. Initially painless, it later caused cosmetic deformity and psychological distress, leading to social withdrawal and poor self-care. Head and neck lipomas represent a small fraction of cases, and those exceeding 10 cm are classified as giant lipomas. Such large lesions may mimic malignancy, delaying diagnosis. Typically, patients present with a progressive, movable, painless swelling, and delayed presentation is common in resource-limited settings, where accurate differentiation remains vital for proper management.

## Introduction

Lipomas are benign tumors arising from adipose tissue, accounting for about 10% of mesenchymal neoplasms [1]. They result from abnormal proliferation or accumulation of adipocytes and are most common in areas rich in fat. Lipomas usually occur between ages 40–50 and are more frequent in males. Around 13% develop in the head and neck region but may also appear in the limbs or trunk [2, 3]. Rarely, they occur in sites such as the anterior neck, infratemporal fossa, or within the oral cavity, pharynx, larynx, or parotid gland. These tumors grow slowly and are usually less than 10 cm, with most measuring about 2 cm [4].

## Case report

A 29-year-old male from rural Northern Gondar, Ethiopia, presented with a posterior neck swelling that had gradually increased in size over three years. Initially lemon-sized and painless, the swelling grew progressively, affecting his daily farming activities and social life. He experienced emotional distress, could not wear regular clothes, and became socially withdrawn. He also reported decreased appetite and unquantified weight loss over two years.

A month before presentation, the mass developed a small ulceration without discharge or fever. At a local clinic, he received ciprofloxacin 500 mg twice daily for seven days with partial improvement, but surgical resources were unavailable. Financial constraints delayed further referral for another month. Eventually, he presented to our hospital for evaluation and possible excision.

**Figure 1:**
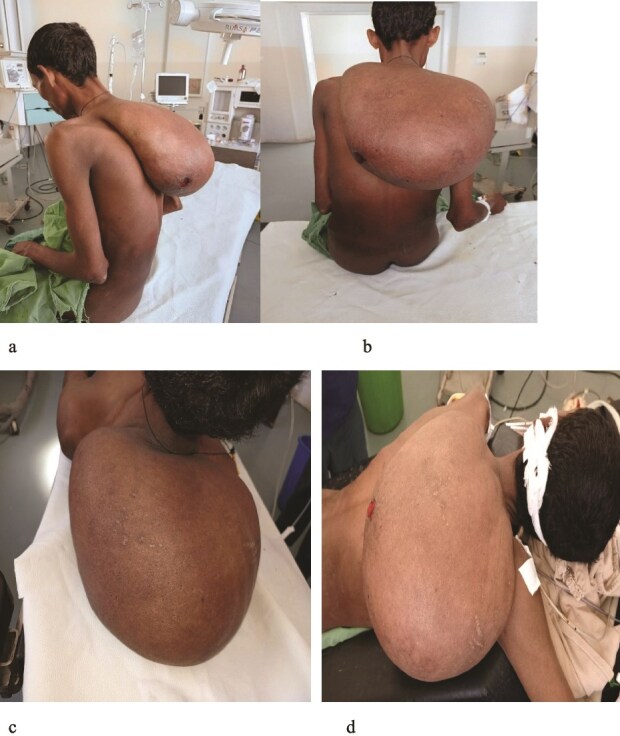
(a–d) Different views of the mass. And 1A MUAC was 15.7 cm.

On admission, he appeared chronically ill and severely emaciated. Vital signs: BP 100/60 mmHg, pulse 87 bpm, respiratory rate 19/min, and temperature 36.4°C. MUAC was 15.7 cm, indicating severe malnutrition (Fig. 1a). A 20 × 25 cm, mobile, non-tender mass arose from the posterior neck and extended toward the upper back (Fig. 1a–d).

**Figure 2:**
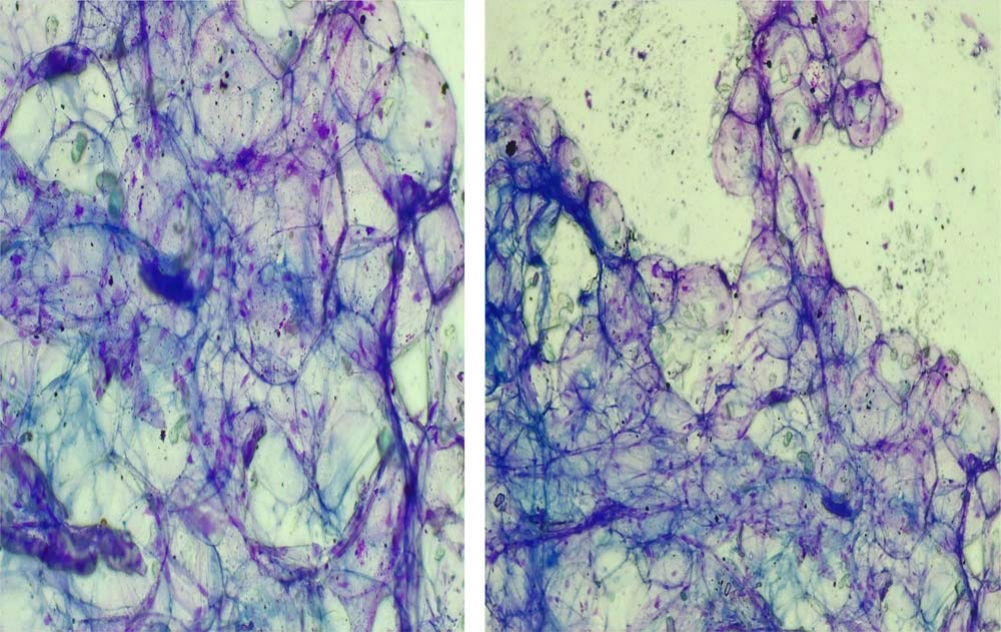
FNAC results showing showed large, round cells with clear cytoplasm.

Investigations revealed: WBC 5.15 × 10^3^/μL, hemoglobin 13.1 g/dL, hematocrit 37%, platelets 162 × 10^3^/μL, and serum albumin 3.0 g/dL. Chest X-ray was normal. Neck ultrasound suggested lipoma and recommended FNAC and biopsy to exclude liposarcoma. FNAC showed large round cells with clear cytoplasm and no atypia or necrosis, consistent with a benign adipocytic lesion (Fig. 2).

**Figure 3:**
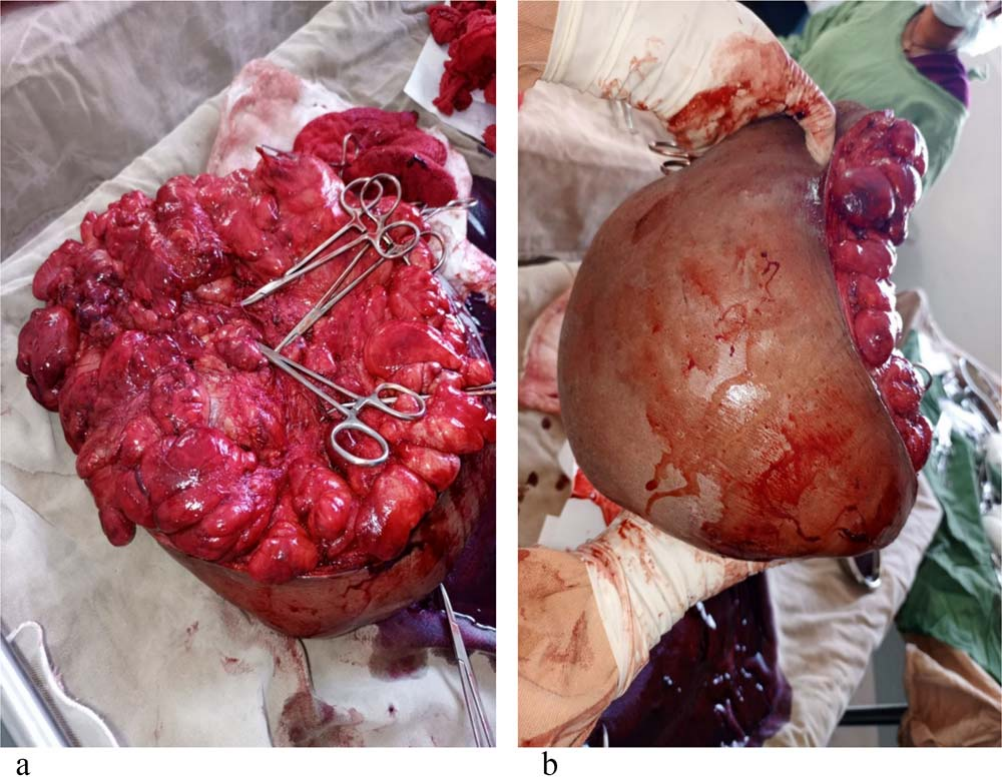
(a, b) The mass after excision (measures around 25 kg).

**Figure 4:**
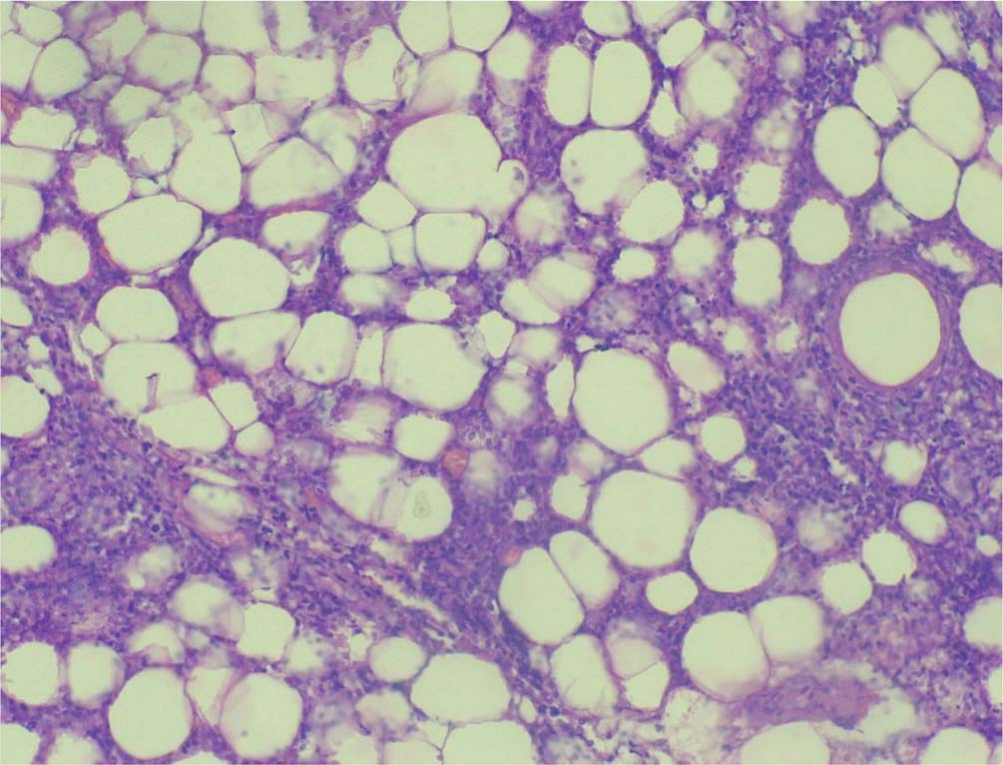
Histopathologic image showing benign adipose tissue tumor.

Surgical excision was performed, and a 25-kg mass was removed meticulously (Fig. 3a and b). Histopathology confirmed a benign adipose tissue tumor (Fig. 4). The patient stayed four days postoperatively, receiving nutritional counseling and hygiene education. On follow-up, he reported full recovery, resumed farming, and regained social confidence.

## Discussion

Lipomas are benign, slow-growing, painless, and mobile tumors, classified by anatomical site and histology. Benign lipomatous tumors include classic lipoma, fibrolipoma, angiolipoma, infiltrating lipoma, intramuscular lipoma, hibernoma, pleomorphic lipoma, lipoblastomatosis, and diffuse lipoblastomatosis [5, 6]. They are either superficial (subcutaneous) or deep (intramuscular) [7].

A lipoma is termed ‘giant’ when exceeding 10 cm or 1000 g [8]. Reports of giant lipomas, particularly in the posterior neck, are rare. Their pathogenesis is unclear, though trauma, Madelung’s disease, dermatophytosis, and endocrine or genetic factors such as Cushing’s disease and HIV-related lipodystrophy have been implicated [9, 10].

Differentiating benign lipomas from well-differentiated liposarcomas can be challenging [11, 12]. Rapid growth, large size, deep location, firmness, or fixation raise suspicion of malignancy. Liposarcomas typically arise in deep tissues—retroperitoneum, buttocks, or lower extremities—and only 2%–8% occur in the head and neck [13–15]. Among subtypes, lipoma-like liposarcoma closely resembles benign lipoma both grossly and microscopically [15].

Computed tomography (CT) and magnetic resonance imaging (MRI) are vital for evaluating neck masses. Simple lipomas appear as well-defined, encapsulated, homogeneous lesions [12, 15]. However, imaging cannot always distinguish lipomas from liposarcomas because thin capsules may appear indistinct, mimicking infiltration [7, 11]. Intralesional components such as fibrous septa, muscle fibers, and blood vessels may further obscure differentiation [12]. Ultrasound, though widely available, is also limited because both lesions can show similar echogenicity [9, 14].

Histological examination remains the diagnostic gold standard. Incisional biopsies may be misleading; definitive diagnosis is usually made after complete excision [14]. Histologically, lipomas consist of mature adipocytes arranged in lobules surrounded by a thin fibrous capsule [10]. Liposarcomas, while similar, show atypical fibroblasts and occasional signet ring cells indicating malignancy [11].

Differential diagnoses include lipoblastoma (infancy), intramuscular angioma, lipomatosis, myxoma, myxoid liposarcoma, and pleomorphic liposarcoma [12]. Accurate histopathologic evaluation is essential for appropriate management.

Surgical excision is the treatment of choice. Traditional excision is preferred over liposuction-assisted removal because it ensures complete removal and lowers recurrence risk. Liposuction may be used selectively to minimize scarring but carries higher recurrence rates and potential complications such as skin dimpling, paresthesia, and pigmentary changes [10, 15]. Simple lipomas recur in about 5% of cases [13], while recurrence of giant lipomas largely depends on completeness of excision.

In our patient, the lipoma qualified as ‘giant,’ measuring 20 × 25 × 15 cm and weighing about 25 kg. Due to unavailability of CT and MRI, diagnosis relied on ultrasound and FNAC, with histopathology confirming the benign nature post-excision. This case underscores that, even in resource-limited settings, timely surgical management can achieve excellent outcomes.

## Conclusion

Lipomas are benign soft tissue tumors that may occur anywhere in the body, but those in the head and neck are uncommon. Because they are usually painless, patients often present late with large swellings. Imaging—ultrasound, CT, or MRI—assists in initial evaluation, but histopathology remains essential for definitive diagnosis. In low-resource settings, limited access to diagnostic tools often delays care. Once diagnosed, surgical excision remains the definitive treatment. In this case, despite limited facilities, the giant posterior neck lipoma was successfully excised, allowing full recovery and improved quality of life. Meticulous surgical removal minimizes recurrence and ensures favorable outcomes.

### Patient’s perspectives

‘I loved my social life and enjoyed working on my farm. But as the swelling on my neck and back grew larger, I stopped attending social gatherings and even stopped enjoying them altogether. I hated going to the farm, and I couldn’t wear the clothes I liked because of the swelling, I even started to avoid foods. I felt so depressed and gave up on everything. But now, I feel like I’ve gotten my old self back. I can enjoy my farm activities again, actively participate in social and community events, and wear the clothes I love clothes I haven’t worn in years. I’m truly grateful to the physicians and healthcare workers who helped me. Thank you.’
